# Evaluating the effects of heat strain and air pollution on kidney health^☆^

**DOI:** 10.1016/j.envint.2025.110042

**Published:** 2025-12-30

**Authors:** Jaime Butler-Dawson, Diana Jaramillo, Lyndsay Krisher, Karely Villarreal Hernandez, Laura Calvimontes, Grant Erlandson, James Seidel, Colton Castro, Stephen Brindley, Yaqiang Li, Miranda Dally, Katherine A. James, Richard J. Johnson, Daniel Pilloni, Alex Cruz, Nicholas Smith, Evan Johnson, Joshua W. Schaeffer, John L. Adgate, Lee S. Newman

**Affiliations:** a Centers for Health, Work, & Environment, Colorado School of Public Health, University of Colorado, Anschutz Medical Campus, Aurora, CO, the United States of America; b Department of Environmental and Occupational Health, Colorado School of Public Health, University of Colorado, Anschutz Medical Campus, Aurora, CO, the United States of America; c Department of Environmental and Radiological Health Sciences, Colorado State University, Fort Collins, CO, the United States of America; d Department of Epidemiology, Colorado School of Public Health, University of Colorado, Anschutz Medical Campus, Aurora, CO, the United States of America; e Division of Renal Diseases and Hypertension, University of Colorado School of Medicine, Anschutz Medical Campus, Aurora, CO, the United States of America; f Pantaleon, Guatemala City, Guatemala; g Independent Researcher, Guatemala; h Division of Kinesiology & Health, University of Wyoming, Laramie, WY, the United States of America; i Division of Pulmonary Sciences and Critical Care Medicine, Department of Medicine, University of Colorado School of Medicine, Anschutz Medical Campus, Aurora, CO, the United States of America

**Keywords:** Heat, Particulate Matter, Kidney, Worker, Climate Change

## Abstract

**Background:**

Rising global temperatures raise concerns about climate change’s health impacts. Agricultural workers are particularly vulnerable due to prolonged exposure to heat and environmental pollutants, including airborne particulate matter (PM), which may increase their risk for kidney injury. This study explores the relationships between core body temperature (CBT), markers of hydration, PM exposure, and kidney injury, offering insights into public health risks related to climate change.

**Methods:**

We conducted a longitudinal study of 148 sugarcane workers in Guatemala, monitoring them over 378 person-workdays. CBT and personal PM exposure were recorded throughout the workday, while markers of hydration and kidney function were assessed pre- and post-shift. Linear mixed-effects models were used to assess the relationships between these exposure factors, dehydration markers, and serum creatinine changes, an indicator of kidney injury.

**Findings:**

CBT exceeded 38.0 °C on 53 % of workdays, with 15 % surpassing 38.5 °C. Higher urinary specific gravity (β: 3.82, 95 % CI: 0.84, 6.80), serum uric acid (β: 2.46, 95 % CI: 0.70, 2.14), average heat index (β: 1.33, 95 % CI: 0.53, 2.14), and CBT exceeding 38.5 °C (β: 8.95, 95 % CI: 2.89, 15.02) were significantly associated with cross-shift increases in serum creatinine. Despite substantial PM_5_ exposure, no significant association with creatinine changes was found.

**Conclusions:**

Heat strain and dehydration are significant contributors to kidney injury in agricultural workers, highlighting critical public health risks. These findings emphasize the urgent need for interventions to mitigate heat-related health hazards, including workplace protections, hydration strategies, and climate adaptation policies to safeguard vulnerable populations worldwide.

## 1. Introduction

As climate change progresses, heat-related morbidity and mortality are expected to rise globally (Bell et al., 2024). Both chronic and acute heat exposure pose significant public health threats, particularly to renal health, as increasing global temperatures push physiological heat tolerance limits (Johnson et al., 2019). In equatorial regions, more frequent and intense heatwaves may contribute to a spectrum of kidney-related health issues, from acute kidney injury (AKI) to chronic kidney disease (CKD) (Ebi et al., 2021).

Epidemiological studies in Latin America have demonstrated that living and working in high ambient temperatures increases the risk of kidney injury and chronic kidney disease of unknown origin (CKDu) (Butler-Dawson et al., 2019; Chapman et al., 2021; Crowe et al., 2010; Garcia-Trabanino et al., 2015). CKDu has also been reported in Mexico, Sri Lanka, India, and Egypt among both worker and community populations. Unlike traditional CKD, CKDu is not associated with established risk factors such as hypertension, diabetes, or advanced age. Although its etiology remains unclear, recurrent heat stress and dehydration are widely hypothesized to be primary contributors.

Sugarcane workers in Latin America face disproportionately high CKDu risk due to the combination of intense manual labor, prolonged environmental heat exposure, dehydration, and fluid loss (Garcia-Trabanino et al., 2015; Petropoulos et al., 2023; Sorensen et al., 2019). Heat stress results from both external environmental heat and internal metabolic heat generated during physical exertion (NIOSH, 2016). Many heat-related studies have relied on ambient weather indices as proxies for heat stress in assessing its relationship with kidney function markers (Butler-Dawson et al., 2019; Garcia-Trabanino et al., 2015; KDIGO, 2013; Butler-Dawson et al., 2021; Hansson et al., 2019). However, few have examined individual-level heat strain—the physiological response to combined environmental and metabolic heat stress—and its direct effects on kidney function. Core body temperature (CBT) is a key measure of heat strain, reflecting an individual’s thermal load and providing insight into the mechanisms of heat-related kidney injury, which remain poorly understood. Given the increasing frequency and severity of heatwaves, elucidating the physiological effects of heat stress is critical for developing strategies to mitigate kidney damage. Additionally, heat stress can impair hydration status, further straining the renal system and exacerbating the adverse health effects of thermal load (Pitts et al., 1944; Snellen, 1966)

Beyond heat stress and dehydration, other environmental exposures—such as particulate matter (PM), pesticides, and heavy metals—may also contribute to kidney injury and CKDu. In Latin America, sugarcane fields are often burned before harvest, exposing workers to high levels of PM (Adgate et al., 2025; Butler-Dawson et al., 2024; Schaeffer et al., 2020). Pre-harvest burning generates airborne pollutants, including silica-dominated particles, metal oxides (e.g., magnesium and iron), and unburned hydrocarbons, some of which have been implicated in kidney disease (Schaeffer et al., 2020; Le Blond et al., 2008; Stem et al., 2024; Rogers et al., 2024; Sasai et al., 2022). Emerging epidemiological evidence suggests a link between PM_2.5_ exposure and kidney disease, particularly in regions with high ambient PM levels (Stem et al., 2024; Rogers et al., 2024; Sasai et al., 2022; O'Brien et al., 1998). However, longitudinal studies on PM exposure among agricultural workers remain limited, and most rely on estimates of ambient air pollution rather than direct personal exposure measurements.

This study aims to investigate the independent and interactive effects of physiological responses to heat stress (i.e., heat strain measured using ingestible gastrointestinal temperature capsules), dehydration, and personal PM exposure on kidney injury among male sugarcane workers in Guatemala.

## 2. Methods

### 2.1. Study details and population

The Chronic Kidney Disease, Heat, and Air Pollution (CHAP I) Study is a prospective, longitudinal study of sugarcane cutters employed at an agribusiness in southwest Guatemala. The field study was conducted across two 6-month harvest seasons (2021–2022 and 2022–2023) through a long-standing collaboration with the company and its clinical staff under a memorandum of understanding with the University of Colorado. All participants provided signed informed consent, and the study was approved by the Colorado Multiple Institutional Review Board (COMIRB) and ZUGUEME Comité Ética Independiente in Guatemala.

Participants were male sugarcane cutters (only males are employed as cutters), aged ≥ 18 years, employed during the study period. Recruitment occurred prior to the start of each harvest season (November 2021 and 2022) and concluded in March or April. Twelve work groups were randomly selected from 24 available groups, and 15 workers from each selected group were randomly recruited and enrolled each season. Work groups typically consist of approximately 50 cutters who follow the same shift schedule. All workers underwent a pre-employment health screening prior to the start of the harvest season. In order to be hired for the season, individuals applying to work the harvest season must have had an eGFR of 60 mL/min/1.73 m^2^.

Cutters harvest recently burned sugarcane using machetes, cutting stalks at ground level, trimming leaves, and piling the cut cane. They work 8–10 h per day, 6 days per week, with three 15-minute breaks and a one-hour lunch. Workers wear long-sleeved shirts, hats, gloves, and boots but no respiratory protection. Additional workplace details have been previously described (Butler-Dawson et al., 2019).

### 2.2. Study design

**Fig. 1.**
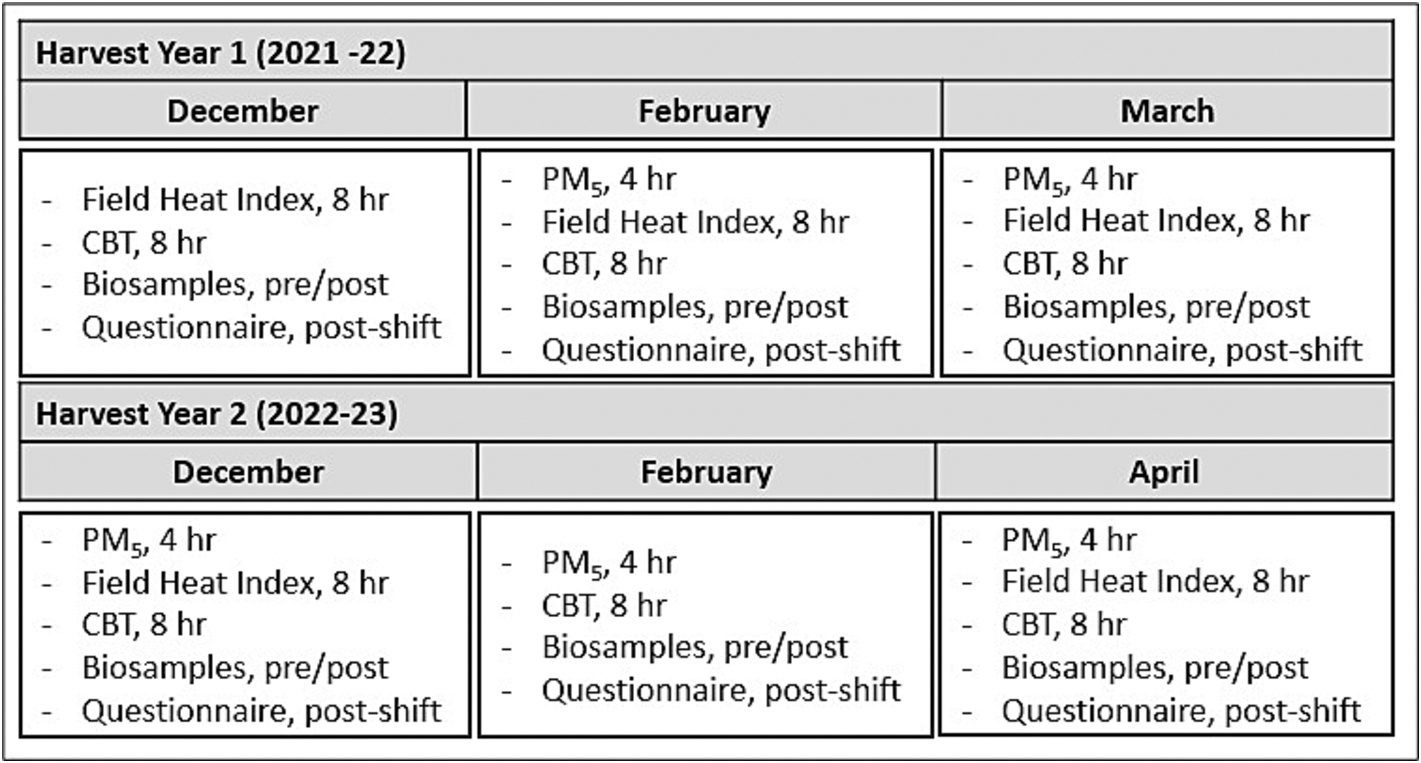
Study Design. Abbreviations: PM_5_, Particulate Matter with a diameter < 5.0 μm; CBT, Core Body Temperature. Pre/Post, pre- and post-shift.

At recruitment, a pre-harvest assessment was conducted to evaluate baseline health, collect urine and blood samples, and administer a questionnaire on demographics and health history. Each worker participated only one harvest season and underwent up to three monitoring periods during the season (December, February, and March/April), approximately one month apart. In each period, we collected repeat personal measures of PM_5_, ambient heat exposure, and individual gastrointestinal temperature during the work shift. Concurrently, we collected pre- and post-shift urine and blood samples and post-shift questionnaire data, Fig. 1.

### 2.3. Exposure monitoring

#### 2.3.1. Ambient heat Exposure

Ambient temperature and relative humidity were recorded daily in the field where the participants worked using the Kestrel 5400 Heat Stress Tracker & Weather Meter (Kestrel Instruments, Boothwyn, PA). The device measured dry-bulb temperature and relative humidity at 15-minute intervals during the entire 8-hour work shift and calculated the heat index (°C). Field weather indices for February 2023 were missing due to technical issues with the weather meter. For each work shift, average and maximum heat index values were summarized.

#### 2.3.2. Particulate Matter Exposure

Personal PM exposure was measured during work shifts across five of six monitoring periods: two in the first harvest (February and March) and three in the second harvest (December, February, and April). A pilot study in December 2021 used a parallel particle impactor (PPI) but was unsuccessful due to filter overloading, leading to the adoption of a cyclone-based sampler. The pilot study established a 4-hour sampling limit to prevent filter overloading, half the typical 8-hour work shift.

During sampling, each worker wore a lightweight, breathable running vest (Ultimate Direction, Model V2, Broomfield, CO) equipped with a personal SKC cyclone sampler, tubing, and pump (SKC Inc., Model AirChek XR5000, Eighty-Four, PA). The cyclone inlet was positioned in the personal breathing zone. Air was drawn at 2 L per minute (L/min), producing a 50 % cut-point at approximately 5 μm aerodynamic diameter (PM5). Filters were 37-mm polyvinyl chloride (PVC) filters, pore size 5.0 μm (SKC Model 225–5-37).

Sampling was monitored by research assistants in the field, and the duration was 4 hrs per shift. PM_5_ time-weighted average concentrations (μg/m^3^) were calculated by dividing filter mass by the sampled air volume. Details of the sampling approach and instrument validation are described in Adgate et al. (2025) (Adgate et al., 2025).

#### 2.3.3. Workday practices

A post-shift questionnaire was administered to collect information on workday practices, including electrolyte intake, number of rest breaks, and tons of sugarcane cut. Participants also reported the source of drinking water used to fill their 5-L container before work, allowing assessment of potential contaminants contributing to nephrotoxicity risk.

#### 2.3.4. Core body temperature (CBT) monitoring

Individual gastrointestinal temperature was measured throughout the work shift using an ingestible telemetric sensor (BodyCap e-Celsius Performance^®^, Caen, France), a validated method for assessing CBT (O'Brien et al., 1998). Workers ingested the pill ~ 10 min before starting work (Notley et al., 2020; Dang and Dowell, 2014), minimizing early excretion risks observed in pilot testing. CBT data were recorded at 2-minute intervals and downloaded via a wireless receiver (e-Viewer, BodyCap, Caen, France).

Due to our study design and missing CBT data, there were 124 workers (84 % of 148) and 223 person-workdays (59 % of 378) in the final CBT data set used for this analysis. Based on study design, up to 12 workers per study day were randomly selected to ingest a pill. There were 91 person-workdays (24 % of 378) where a worker was not selected to ingest a pill and thus had no CBT data. In terms of missing data, several person-workdays had missing CBT data either in the morning or afternoon, however we could not confirm whether the pill had been excreted or if there were technical issues (i.e., a synchronization problem between the monitor and the pill). Person-workdays that were missing greater than 30 % of the CBT values or had seemingly non-random missingness of CBT values (i.e. missing data were identified either all in the morning or afternoon) were excluded from the CBT dataset. There were 64 person-workdays (17 % of 378) that were excluded from the final CBT dataset due to missing data. The first 30 min of data were removed to account for capsule transit time (Egbert et al, 2022; Hertzberg et al., 2017). Clinical and kidney function markers were compared between excluded (due to missingness) and included workers, showing no significant differences in age, BMI, blood pressure, hydration markers, or creatinine levels (pre-shift, post-shift and change in creatinine).

To quantify the CBT response, we identified the maximum CBT (°C) for each person-workday and calculated the net area under the curve (AUC) for CBT across the 8-hour work shift (Horswill et al., 2008; Datta et al., 2021). The AUC was calculated using the composite trapezoid rule (AUC function in the DescTools package, R version 4.4.1) to estimate the dynamic thermal response to thermal load.

We defined occupational heat strain based on CBT thresholds, increased CBT: 38.0 – <38.5 °C (100.4 – <101.3 °F) or extreme CBT: 38.5 °C or greater (≥101.3 °F). These thresholds align with the U.S. National Institute for Occupational Safety and Health (NIOSH) heat stress risk guidelines and the American Conference of Governmental Industrial Hygienists (ACGIH) recommendations, which suggest that CBT should not exceed 38.0 °C for non-acclimatized workers and 38.5 °C for acclimatized workers to prevent heat exhaustion or stroke (ACGIH, 2009; ISO, 2004; Coco et al., 2016).

#### 2.3.5. Urine and blood biomarkers

The primary outcome was the percent change in serum creatinine from pre-shift to post-shift ([post-shift – pre-shift / pre-shift] x 100), individually calculated at all the monitoring periods. Blood and urine samples were collected by a trained phlebotomist before and after each monitoring workday. A venous whole blood sample was analyzed immediately using the iSTAT Handheld Blood Analyzer (Abbott Point of Care Inc., Princeton, NJ) with a Chem8 + cartridge, measuring creatinine, sodium, potassium, glucose, and blood urea nitrogen.

Urine analysis included measuring urine specific gravity urine specific gravity (USG) in the field using a digital refractometer (ATAGO PAL-10S, Tokyo, Japan) and performing urinalysis within two hours using Mission Urinalysis Reagent strips (ACON Laboratories, San Diego, CA, USA). Remaining samples were stored at −20 °C and sent to an independent laboratory (Herrera Llerandi Laboratory, Guatemala City) for biochemical analysis, including creatine phosphokinase, uric acid, and osmolality. Serum creatinine was used to estimate eGFR using the 2021 CKD Epidemiology Collaboration formula (Inker et al., 2021).

To assess hydration status, which lacks a universal gold standard, we examined several markers at both pre- and post-shift time points (Armstrong, 2007). These markers included USG, serum osmolality, urine osmolality, serum uric acid, and blood sodium. In addition, we calculated the fractional excretion of sodium (FENa) and the fractional excretion of uric acid (FEUA) using standard methods (Steiner, 1982; Stibůrková et al., 2006). FENa provides insight into tubular sodium handling and helps differentiate decreased kidney perfusion (FENa < 1 %, often due to dehydration) from intrinsic kidney damage (FENa ≥ 1 %) (Seethapathy and Fenves, 2022). FEUA indicates the efficiency of uric acid reabsorption and excretion and assists in distinguishing between renal and prerenal acute kidney failure.

For descriptive purposes and to support evidence of dehydration and urine concentration, we categorized hydration markers as follows: USG (hydrated < 1.020 vs. dehydrated ≥ 1.020), blood sodium (hyponatremic < 135 mEq/L vs. normal ≥ 135 mEq/L), FENa (<1.0 % vs. ≥ 1.0 %), and FEUA (<10.0 % vs. ≥ 10.0 %). Additionally, urinary pH and leukocyte esterase were assessed using dipsticks. Urinary pH was categorized as acidic (5.0), neutral (6.0–6.5), or alkaline (≥7), and leukocyte esterase was categorized as normal (<70 Leu/μL) versus high (≥70 Leu/μL).

#### 2.3.6. Questionnaire and clinical data

The pre-harvest questionnaire collected data on personal and household characteristics, medical history, lifestyle behaviors, medication use, and work history using a previously established questionnaire (Butler-Dawson et al., 2019; Butler-Dawson et al., 2018). Pre-harvest clinical assessments included orthostatic and seated blood pressure, body mass index (BMI), hemoglobin A1c (HbA1c), and anthropometric measurements. Body composition was assessed using skinfold caliper measurements (chest, triceps, and subscapular) by trained field staff (Creative Health Slim Guide, Plymouth, MI, USA), and body density, fat mass, body fat percentage, and fat-free mass (an estimate of muscle mass) were calculated.

During all the monitoring sessions, pre-shift blood pressure was measured after at least three minutes of seated rest (OMRON Healthcare, Inc., Lake Forest, IL). The post-shift questionnaire gathered information on individual behaviors, including cigarette consumption since waking and use of oral and parenteral medications, vitamins, and supplements in the past 24 hrs. As in a prior study participants were shown images of locally available medications to aid identification (Butler-Dawson et al., 2019). We categorized responses to the medicine use questions, as non-steroidal anti-inflammatory drugs (NSAIDs) use (yes/no) and antipyretic use (yes/no) based on the names of drugs reported. NSAID types included ibuprofen, aspirin, diclofenac, naproxen, or local brands that were identified as an NSAID. Antipyretics include all NSAIDs plus acetaminophen brands.

#### 2.3.7. Statistical analysis

Descriptive data were summarized using medians, interquartile ranges, and frequencies. Differences between pre- and post-shift biomarker values (hydration and muscle damage markers) were assessed using paired t-tests. To estimate the effects of workday practices, hydration markers, and heat exposure on percent change in creatinine, we developed three linear mixed-effects models guided by directed acyclic graphs (DAGs).

To minimize potential confounding due to differing workday practices and exposures between the work groups, we first examined the impact of these practices and exposures on percent change in creatinine using a linear mixed effect model while controlling for work group, with fixed effects for work shift duration, tons of sugarcane cut, number of rest breaks, number of electrolyte packets, morning drinking water source, personal PM_5_ concentrations and field heat index values. All models included a random intercept by individual. Variables with P < 0.05 were retained in the multivariable model (Model 1).

Next, we examined the impact of physiological markers and individual lifestyle factors on percent change in creatinine using univariate linear mixed effect models. Based upon the literature, we selected risk factors *a priori* that we hypothesized would be associated with markers of kidney injury. Significant variables (P < 0.05) were included in a multivariable model (Model 2) alongside average heat index (the only significant variable from Model 1) while controlling for age. To measure the effect of work group, our multivariable model included a random effect for individual and for work group to account for random variation within and between individuals as well as work groups.

Finally, for Model 3, we included CBT. We did not include CBT in Model 2 due to the reduced number of CBT measures. Since CBT category and average heat index were not collinear, both were retained in the model. We used the CBT category variable instead of maximum CBT to examine exposure–response trends. Results for all the models are presented as percent change in creatinine and 95 % confidence intervals (CI). We examined interactions between PM, heat index, CBT indices and markers of hydration based on *a priori* assumptions. Collinearity among exposures and risk factors was evaluated using correlation matrices and variance inflation factors (VIF) in multivariable models. All statistical analyses were performed using SAS version 9.4 (Cary, NC) with two-sided tests (alpha = 0.05).

## 3. Results

### 3.1. Worker Characteristics, Lifestyle behaviors and workday practices

Table 1 illustrates the pre-harvest characteristics of the study population. A total of 148 workers participated, contributing 378 person-workdays across two harvest seasons. Almost 70 % of the participants were from the Department of Escuintla. The average age was 29 years, ranging from 18 to 57. At pre-harvest, nine workers (6 %) were classified as having Stage 2 Hypertension, defined as a systolic blood pressure of 140 mmHg or higher and/or a diastolic blood pressure of 90 mmHg or higher. The median HbA1c level was 5.0 %, with five workers in the prediabetic or diabetic range (≥ 5.7 %). Median estimated glomerular filtration rate (eGFR) at baseline was 120.6 ml/min/1.73 m^2^ and 17 workers (12 %) had an eGFR < 90 ml/min/1.73 m^2^. Almost one quarter (22 %) of the workers reported smoking daily or less than daily at pre-harvest. Use of antipyretic medication during the harvest was common, reported by 66 % of workers, where the use of NSAID medications was less frequent, with 28 % reporting use.

Table 2 presents descriptive statistics for all 378 person-workdays, including workday practices, daily exposures, and biomarker levels. The median shift duration was 9.5 h, with workers reporting a median of three self-reported rest breaks per shift. On average, workers cut five tons of sugarcane per day, with a range of two to nine tons. Two-thirds of person-workdays (67 %) reported using well water as the first drinking water source before coming to work, compared to 27 % who used municipal water and 6 % who relied on other sources, such as purchased bottled water or field tank water.

### 3.2. Exposures

**Fig. 2.**
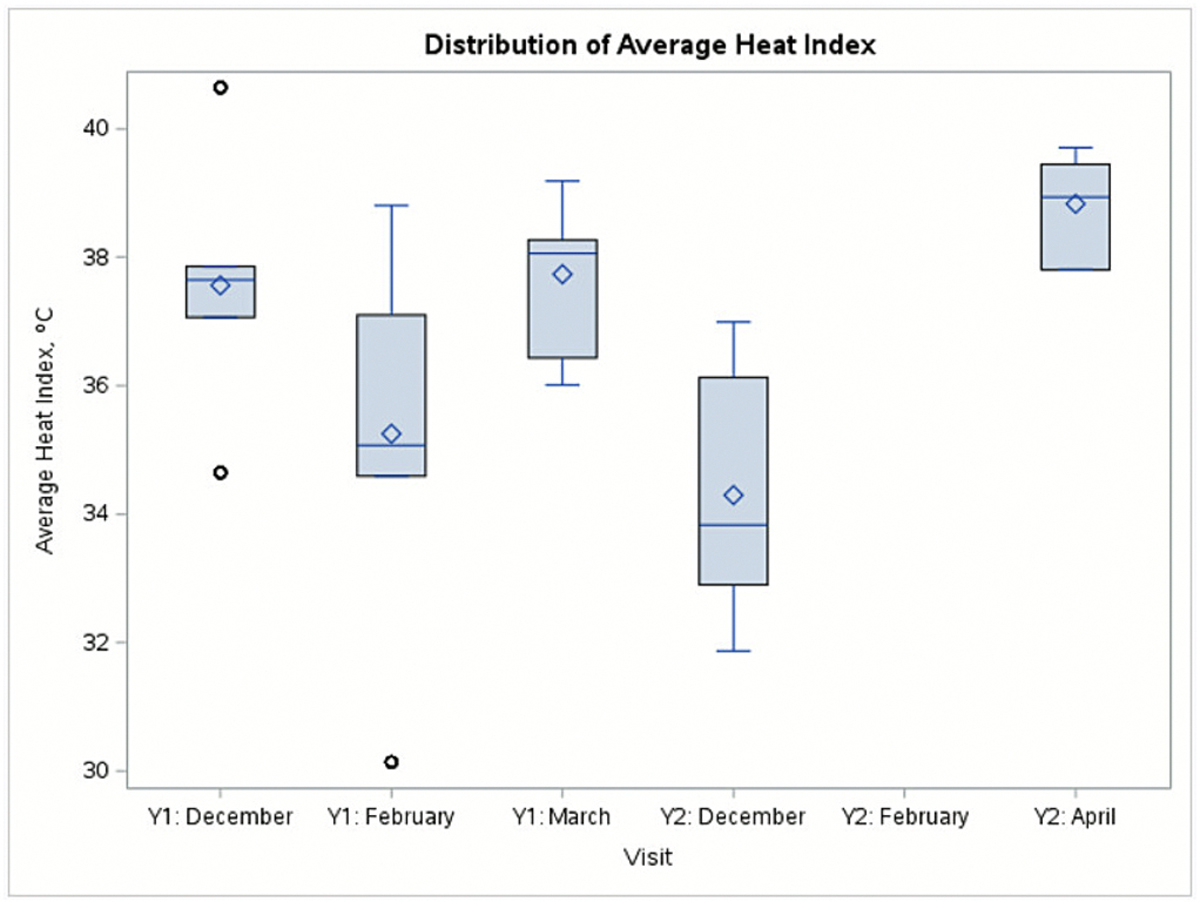
Distribution of average heat index across the six study periods. In February of Year 2, field heat index data were not collected due to technical issues with the WBGT meter. Y1 = Year 1; Y2 = Year 2.

Workers were exposed to high temperatures, with a median average field heat index levels of 37.8 °C (range: 30.1 to 40.6 °C) and a median maximum field heat index of 46.4 °C (range: 39.4 to 60.1 °C) (Fig. 2). On all monitoring days, the median maximum heat index values met the U.S. Occupational Safety and Health Administration’s “High” and “Very High to Extreme” heat risk thresholds (OSHA, 2017).

**Fig. 3.**
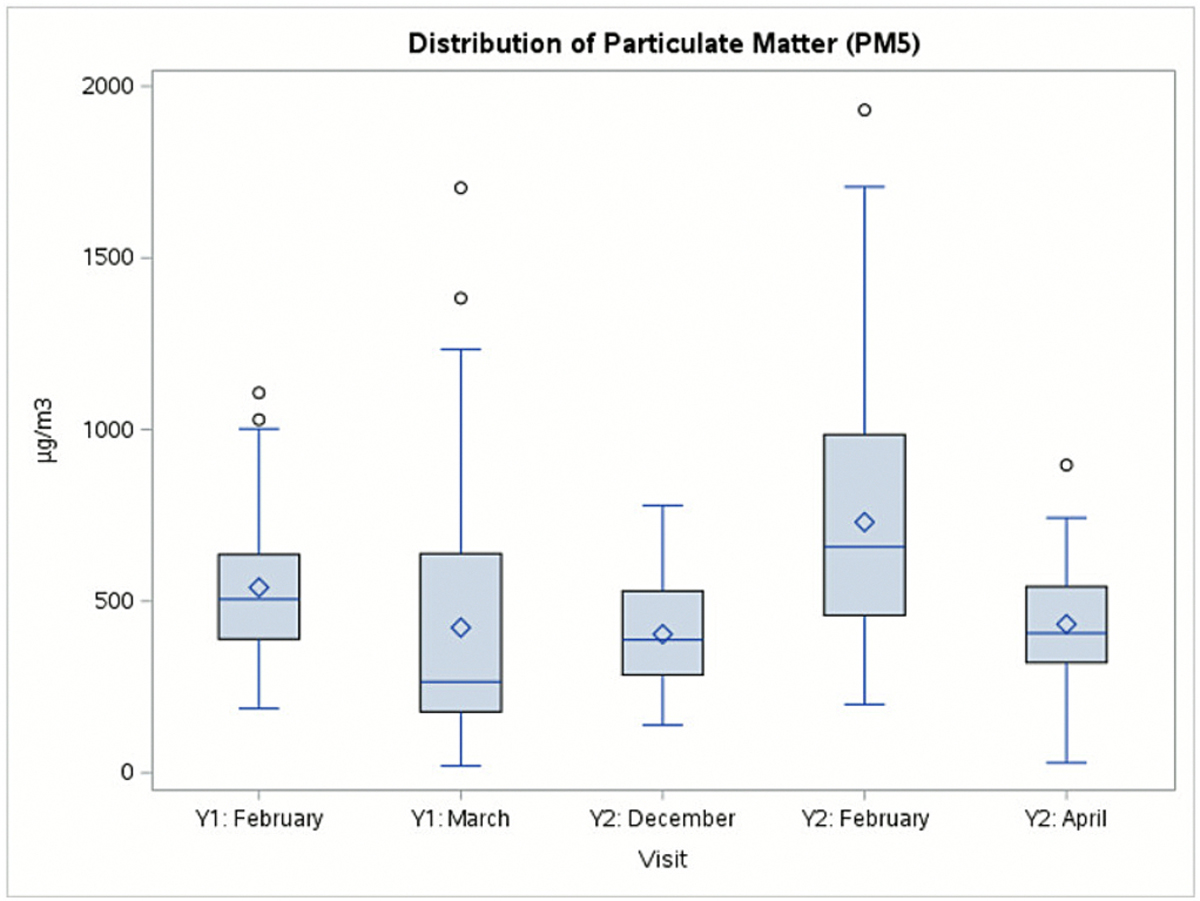
4-hour average personal PM5 measurements (μg/m^3^) by sampling visit. Y1 = Year 1; Y2 = Year 2.

Personal PM_5_ exposure was assessed across 285 person-workdays over 29 sampling days. The 4-hour PM_5_ concentrations were highly skewed with substantial day-to-day variability, with daily median PM_5_ concentrations ranging from 210.4 to 1024.7 μg/m^3^ and an overall median of 448.5 μg/m^3^ (Fig. 3).

### 3.3. Core body temperature

**Fig. 4.**
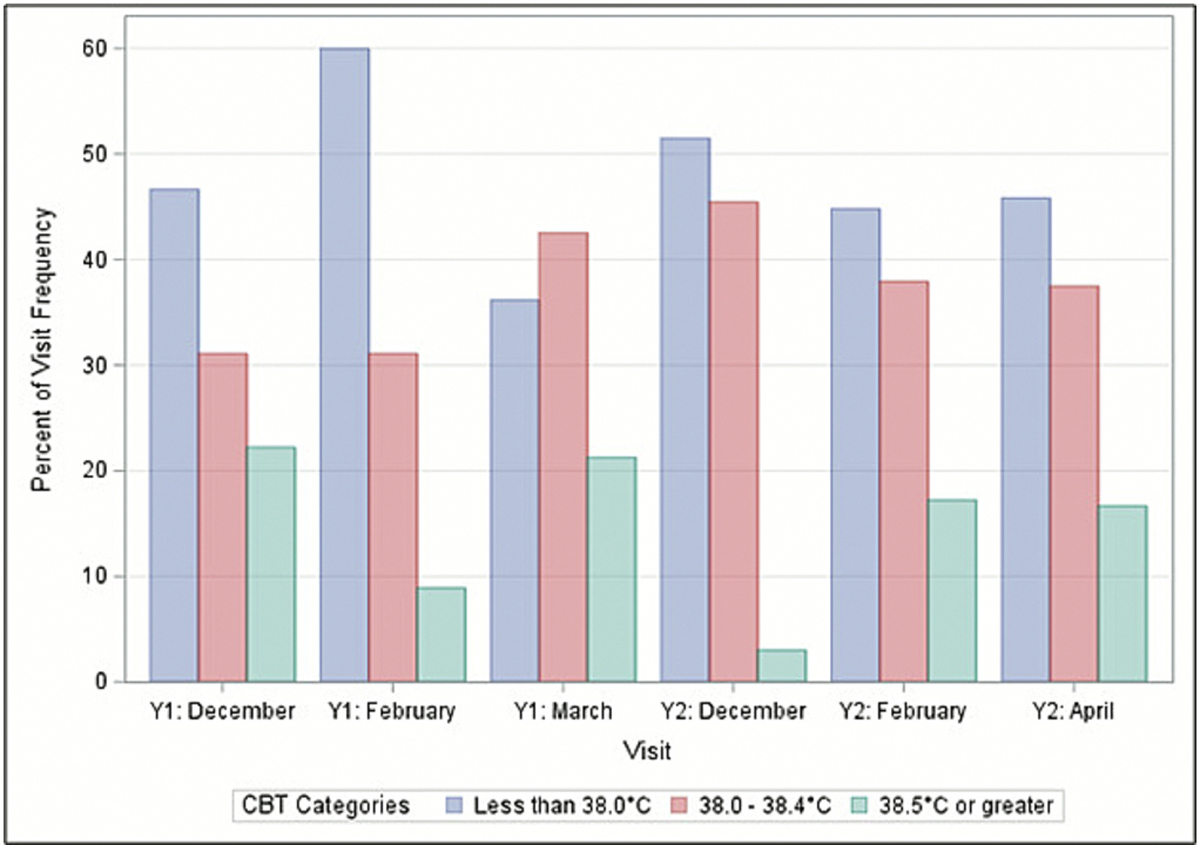
Worker's core body temperature (CBT) during the workday for each study month. Y1 = Year 1; Y2 = Year 2.

The median maximum CBT was 38.0 °C (interquartile range (IQR): 37.8–38.3 °C) across 223 person-workdays with CBT data. CBT reached or exceeded 38.0 °C (100.4 °F) on 53 % of these workdays (n = 117), and 15 % (n = 34 person-workdays) reached 38.5 °C or greater (15 %). Fig. 4 illustrates the distribution of CBT categories by visit month. Among workers with CBT data, 68 % (n = 84) experienced at least one workday with CBT ≥ 38.0 °C or greater on at least one workday, while 23 % (n = 29) had at least one workday with CBT ≥ 38.5 °C.

### 3.4. Markers of Hydration, muscle Breakdown, and Urinalysis results

As shown in Table 2, workers were generally well-hydrated before and after shifts based on median urine specific gravity (USG), serum and urine osmolality, and serum uric acid. However, 12 % of workers began the shift dehydrated (USG ≥ 1.020), which decreased to 7 % post-shift. Hypo-osmolality was common, with 50 % of person-workdays showing post-shift serum osmolality < 280 mmol/kg.

Several markers of hydration significantly declined from pre- to post-shift, including USG (t-value = 7.37, p < 0.01), serum osmolality (t-value = 7.81, p < 0.01), urine osmolality (t-value = 7.29, p < 0.01), blood uric acid (t-value = 2.79, p = 0<.01) and blood sodium (t-value = 14.96, p = 0<.01). Despite this, the prevalence of hyponatremia increased from 3 % pre-shift to 21 % post-shift. FENa was ≥ 1.0 % in 51 % of person-workdays, and 10 % of workers had a post-shift FEUA ≥ 10.0 %.

Urinalysis showed low rates of post-shift aciduria (14 %) and positive leukocyte esterase (2.4 %). Creatine kinase levels significantly increased from pre- to post-shift (324.0 and 448.0 U/L, t-value = 364, p < 0.01), indicating muscle breakdown.

### 3.5. Markers of kidney function

**Fig. 5.**
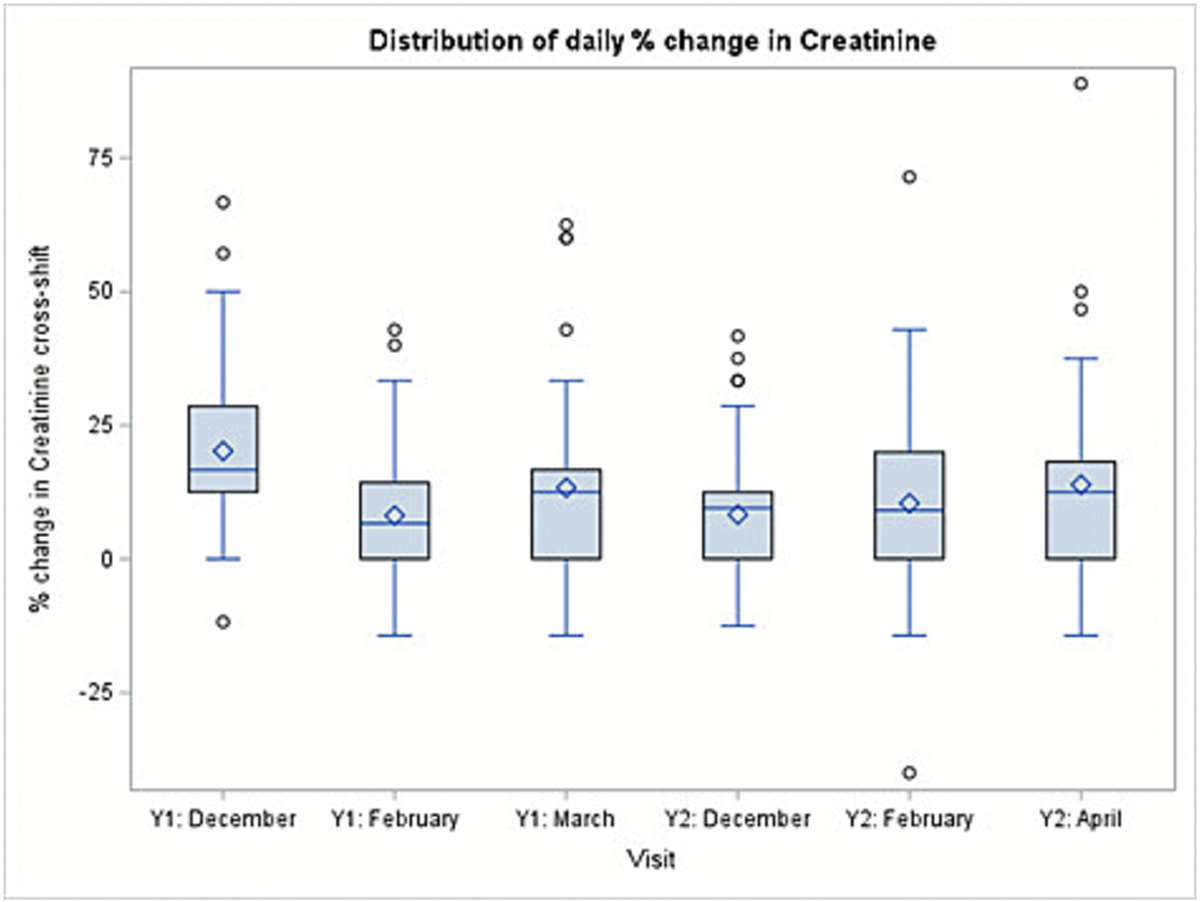
Distribution of percent change in creatinine across the six study periods. A positive percent change indicates a decline in kidney function from pre- to post-shift, N = 148 sugarcane cutters with 378 observations. Y1 = Year 1; Y2 = Year 2.

The primary outcome, percent change in serum creatinine, had a median increase of 12.5 % across 35 monitoring days. The highest median increase occurred in December 2021, with monthly medians ranging from 7.8 % to 14.6 % (Fig. 5). Post-shift creatinine levels were significantly elevated compared to pre-shift values (t-value = −15.41, p < 0.01). AKI, defined as an increase in creatinine of ≥ 0.3 mg/dL or ≥ 1.5 times the pre-shift value (KDIGO, 2012), was observed in 35 person-workdays (9.5 %).

### 3.6. Factors associated with percent change in creatinine

In Model 1, which assessed workday practices and exposures (Table 3), only the average heat index remained significantly associated with an increase in percent change in creatinine (β: 1.17, 95 % CI: 0.36, 1.97). Other factors, including work shift duration, sugarcane tons cut, and number of rest breaks, were not significant. Notable, personal PM_5_ exposure was not associated with changes in creatinine (β: −0.004, 95 % CI: −0.01, 0.003).

Markers of dehydration, including increasing blood sodium, increasing USG, increasing blood uric acid, and decreasing FENa and FEUA, were associated with greater percent changes in creatinine (Table 4). Pre-shift blood sodium, post-shift USG, and post-shift serum uric acid were selected for the second multivariable model (Model 2) based on model fit. Lifestyle factors and creatine kinase levels (pre-shift, post-shift, and cross-shift changes) were not significantly associated with creatinine change.

In Model 2, controlling for age and work group and including heat index from Model 1, significant associations remained for pre-shift blood sodium (β: 0.72, 95 % CI: 0.02, 1.42), post-shift USG (β: 3.38, 95 % CI: 0.99, 5.76), post-shift serum uric acid (β: 2.11, 95 % CI: 0.77, 3.45), and average heat index (β: 1.21, 95 % CI: 0.59, 1.82) (Table 5).

In Model 3, the CBT categorical variable was added to Model 2 (Table 5). Post-shift USG, post-shift serum uric acid, and heat index remained significant, with similar effect estimates. Workers with a CBT of 38.5 °C or greater had a significantly higher percent change in creatinine (β: 8.95, 95 % CI: 2.89, 15.02) compared to those whose CBT remained below 38.0 °C.

Exploratory analyses examining interactions between PM_5_, CBT, heat index, and hydration markers did not identify any significant synergistic effects.

## 4. Discussion

Our results provide critical insights into the prevalence and severity of heat strain, a physiological response to metabolic and environmental heat stress, and its association with kidney injury, as evidenced by cross-shift increases in serum creatinine. With global warming intensifying heat-related risks, these results have substantial public health implications, particularly for vulnerable populations working in hot environments.

Over half of the 223 person-workdays in our study recorded CBT exceeding 38.0 °C, with one-quarter of workers reaching 38.5 °C or higher. This suggests that heat strain is widespread among agricultural workers in hot climates, underscoring the need for targeted health interventions. Globally, an estimated 42 million workers in regions such as Central America, India, and Sri Lanka, along with approximately 2.3 million workers in the U.S., are exposed to occupational heat stress (Chapman et al., 2021) and may be at heightened risk for kidney injury. There is increasing evidence from mechanistic and occupational studies that recurrent kidney injury contributes to the development of CKDu (Dally et al., 2020; Kupferman et al., 2018; He et al., 2017). The International Labour Organization further estimates that 2.41 billion workers worldwide are at risk of excessive heat exposure (Flouris et al., 2024). With climate change contributing to more frequent and intense heatwaves, we anticipate an increase in occupational heat strain, kidney damage, and other heat-related health issues. These findings underline the urgent need for policies and interventions tailored to local conditions that protect workers and mitigate the impacts of climate change.

Our study demonstrates that excessive heat strain is common among sugarcane workers, placing them at increased risk of kidney injury. By continuously measuring gastrointestinal temperature, we assessed the intensity of heat strain experienced by individual workers, providing a more accurate estimate of their response to exertional heat stress. Among CBT metrics, maximum CBT, rather than AUC, was significantly associated with kidney injury, suggesting that brief temperature exceedances may be more predictive of risk than cumulative heat load. Maximum CBT may also be more relevant for workplace policies that trigger immediate interventions once a worker’s temperature surpasses a critical limit. Telemetric gastrointestinal temperature measurements are comparable to esophageal and rectal readings during physical activity in hot environments (Casa et al., 2007). Our findings, in conjunction with other studies, emphasize the need for further investigation into work-rest cycles and preventative strategies during occupational heat exposure (Water, 2016; OSHA, 2017). The International Organization for Standardization (ISO) defines the upper limit of the acceptable rectal temperature limit as 38.0°C (Sakoi et al., 2024), while the ACGIH sets the limit at 38.0°C for unacclimatized workers and 38.5°C for acclimatized workers (ACGIH, 2009). The ACGIH asserts that a core body temperature exceeding 38.5°C (101.3°F) poses a risk for heat-related illnesses. In this current study, 15% of person-workdays with sufficient data reached or surpassed this threshold. Comparatively, a study in California found that 8.3% of Latino farmworkers reached a CBT of at least 38.5°C (Mitchell et al., 2017), and in Florida, fernery workers' CBT exceeded 38.0°C on 57% of their workdays (Mac et al., 2019). Our finding of 53% of person-workdays exceeding 38.0°C aligns with these studies, underscoring the urgency for interventions addressing heat risks for workers in hot environments.

The activation of the sympathetic nervous system is believed to be a main mechanism through which heat stress stimulates renal vasoconstriction (Wilson, 2017). During heat stress, blood is redistributed from the kidneys to prioritize blood flow to the skin and active skeletal muscles, leading to reduced renal blood flow. This redistribution can result in localized renal ischemia and hypoxemia, which may deplete adenosine triphosphate (ATP) and promote oxidative stress and inflammation in the kidneys, ultimately increasing the risk of AKI (Rashid et al., 2024). Similar relationships between CBT and kidney injury have been observed in both field-based and laboratory studies (Petropoulos et al., 2023; Moyce et al., 2017; Roncal-Jimenez et al., 2018; Sato et al., 2019; Chapman et al., 2020; Schlader et al., 2017). One study suggested that peak core temperature effectively distinguishes individuals at risk for AKI (Chapman et al., 2024). Moyce et al. (Moyce et al., 2017) found that hyperthermia increased the risk of AKI among agricultural workers in California. Similarly, Junglee et al. (2013) observed that a 1.3°C rise in core temperature, along with approximately 1% body weight loss, resulted in elevated serum creatinine and reduced urine flow rate (Junglee et al., 2013). Schlader et al. (2017) reported that longer durations of exercise in the heat, which lead to greater increases in core temperature and more significant dehydration, were associated with higher serum creatinine levels (Schlader et al., 2017). Additionally, we observed that elevated serum uric acid levels correlated with increased creatinine, which is consistent with findings from previous studies. Heat stress is known to raise serum uric acid levels, partially due to reduced renal blood flow (Knochel et al., 1974). Our study also found an increase in creatine kinase levels during the work shift, likely due to muscle breakdown from the physical exertion of cane cutting. However, no significant association was found between creatine kinase levels and changes in creatinine (Butler-Dawson et al., 2019; Sorensen et al., 2019). Thus, our findings suggest that elevated CBT is a key factor of kidney injury mechanisms among sugarcane workers, reinforcing the need for interventions aimed at reducing heat exposure and mitigating its harmful effects.

Several markers of dehydration were identified as risk factors for increased creatinine during the work shift, suggesting that elevated core body temperatures, indicative of heat strain, combined with dehydration, can significantly impact kidney function (Chapman et al., 2020). Dehydration impairs thermoregulation, leading to higher core temperatures. It also reduces blood volume (hypovolemia), triggering the release of vasopressin, aldosterone, and renin, which further decreases renal blood flow. This cascade promotes sodium reabsorption, increasing urine concentration. As thermoregulation is impaired, core temperatures rise (Sawka et al., 1998). Notably, no association was found between serum osmolarity and percent change in creatinine, suggesting that hypovolemia, rather than hyperosmolarity, may play a greater role in the creatinine increases observed. The absence of hyponatremia at the start of the shift indicates that sodium and volume regulation were adequate prior to work (Chapman et al., 2021). However, post-shift, 21% of person-workdays showed hyponatremia, which likely reflects sodium loss through sweat combined with fluid replacement low in electrolytes rather than overhydration. Together with the low FENa values, these findings point to dehydration and sodium depletion as concurrent contributors to kidney stress during heat exposure.

Another notable finding was the significant association between lower FEUA and FENa and increased creatinine. While most workers maintained adequate hydration, the FENa values suggest that dehydration, leading to reduced kidney perfusion, may have contributed to the observed rise in creatinine. FENa, calculated from blood and urine samples, serves as an indicator of the renal response to changes in volume status and blood flow. These findings are consistent with Omassoli et al. (2019), which identified a post-exercise link between lower FENa and AKI, indicating pre-renal kidney function reduction (Omassoli et al., 2019). Similarly, a study of marathon runners found that lower FENa values reflected dehydration-induced decreased kidney perfusion, contributing to increased creatinine levels (Mansour et al., 2017).

As previously reported by our team (Adgate et al., 2025), personal breathing zone PM_5_ samples collected from the workers demonstrated consistently elevated concentrations (~450 μg/m^3^) (Adgate et al., 2025). While detailed spatial and temporal patterns of PM exposure were described in the earlier work (Adgate et al., 2025), this present study emphasizes the relevance of these elevated exposures to kidney health outcomes. The high particulate burden likely reflects constant resuspension of burnt sugarcane ash and dust while cutting.

Emerging evidence suggests that air pollution may exacerbate the effects of climate-related heat stress (Alisoltani et al., 2024; Chen et al., 2024). For instance, one study found that higher ambient temperatures significantly exacerbated the estimated impact of PM_2.5_ and its components on CKD hospitalizations (Wang et al., 2024). Mechanistically, concurrent exposure to heat and PM may contribute to oxidative stress, systemic inflammation, and reduced renal perfusion. Understanding the combined effects of heat and air pollution on renal function is therefore critical, as both stressors may act synergistically to accelerate kidney injury (Rahman et al., 2022; Chowdhury et al., 2024). Future studies by our team will build on this work to examine heat-PM interactions and characterize the nephrotoxic constituents of PM to clarify their joint impact on kidney function.

While this study offers valuable insights into the variability and patterns of heat strain among sugarcane cutters, several limitations may affect the generalizability of the findings. One key limitation is the inclusion of only trained male workers, as hypohydration may impact female workers and untrained individuals differently. Thus, the findings may not fully capture these variations. Additionally, approximately 17 % of CBT data were missing. If this data loss resulted from technical issues with CBT sensors, any resulting bias would likely lead to non-differential misclassification of the outcome, potentially attenuating the strength of the findings.

To mitigate occupational heat strain, adaptation and prevention measures focus on water, rest, and shade (WRS) practices, with attention to feasibility in field conditions. However, few systematic studies assess their effectiveness (Chicas et al., 2021; Sankar et al., 2024; Wegman et al., 2017; Hansson et al., 2024). Notably, a recent WRS intervention study showed persistent cross-shift kidney dysfunction and systemic inflammation that worsened throughout the workweek, despite the intervention (Lucas et al., 2025). Independent or complementary strategies, such as enhanced hydration, adjusted work intensity, shift rotations, and more frequent breaks, may help reduce heat strain when self-pacing is not feasible due to wage structures (Miller et al., 2011).

## 5. Conclusion

Our findings indicate that a considerable proportion of sugarcane workers reach or exceed the recommended CBT limits, leading to cross-shift declines in kidney function. These results underscore the need for continued research into human thermoregulation under heat stress and for intervention strategies, such as more frequent breaks, post-heat recovery protocols, and hourly pay adjustments, to reduce heat strain, dehydration, and kidney injury in agricultural workers.

## Figures and Tables

**Table 1:** Pre-harvest characteristics among 148 male sugarcane cutters across two harvest seasons.

Pre-harvest (November 2021 and 2022)	Characteristics, median (IQR), n (%)

Age, years	29 (24, 38)
Hypertension*, n (%)	9 (6 %)
Body Mass Index, kg/m^2^	22.6 (21.3, 24.3)
Fat Free Mass	52.48 (48.12, 56.59)
Fat Mass	6.65 (4.47, 9.76)
HbA1c, %	5.0 (4.7, 5.2)
Blood Glucose, mg/dL	97.0 (90.0, 102.5)
Hematocrit, %	44 (43, 47)
Hemoglobin, g/dL	15.0 (14.6, 16.0)
**Survey Data**	
Number of previous harvests worked	8 (5, 16)
Family history of kidney disease, n (%)	2 (1 %)
NSAID use, n (%)	106 (28 %)
Antipyretic use, n (%)	238 (63 %)
Smoking Status, n (%)	
- Current (daily or less than daily)	15 (22 %)
- Past	17 (25 %)
- Never	37 (54 %)
Municipality	
- Escuintla	102 (69 %)
- Suchitepéquez	42 (28 %)
- Other**	4 (3 %)
Race	
- Ladino	98 (66 %)
- Maya / Indigena	21 (14 %)
- Garifuna	4 (3 %)
- Other	25 (17 %)
**Markers of Kidney Function**	
Creatinine, mg/dL	0.8 (0.7, 0.9)
eGFR, ml/min/1.73 m^2^	120.59 (108.49, 128.33)
Kidney Disease Stages, ml/min/1.73 m^2^, n (%)	
- eGFR > 90	124 (88 %)
- eGFR 60–89	15 (11 %)
- eGFR < 60	2 (1 %)

* Hypertension, defined as systolic blood pressure ≥140 mmHg and/or diastolic blood pressure ≥90 mmHg.

eGFR: estimated glomerular filtration rate.

** "Other” includes Chimaltenango, Guatemala, and Quiche departments.

**Table 2:** Characteristics for all monitoring days (378 person-workday) among the 148 sugarcane cutters across two harvest seasons.

Characteristics	Median (IQR)

Systolic blood pressure, mmHg	118 (108, 123)
Diastolic blood pressure, mmHg	67 (63, 74)
**Workday Practices and Exposures**	
Work shift duration, hours	9.5 (9.2, 9.9)
Self-reported number of tons cut (n = 329)	5 (5, 6)
Self-reported number of rest breaks	3 (3, 4)
Number of electrolyte packets, 1-L packets	5 (5, 5)
Morning Water Source for 5-L Thermos, n (%)	
- Well	250 (67 %)
- Municipal	103 (27 %)
- Other (i.e., Field Tank and Bought Water)	22 (6 %)
Particulate Matter, PM_5_, μg/m^3^ (n = 285, 75 %)	448.5 (298, 636)
Average field heat index, °C	37.8 (37.1, 38.9)
Maximum field heat index, °C	46.4 (43.5, 50.3)
**Core Body Temperature (n = 223, 59 %)**	
CBT Categories, n (%)	
- Normal, less than 38.0 °C	106 (48 %)
- Increased, 38.0 – 38.4 °C	83 (37 %)
- Extreme, 38.5 °C or greater	34 (15 %)
Maximum CBT, °C	38.0 (37.8, 38.3)
**Physiological Markers, Pre- and Post-Shift**	
Pre-shift urine specific gravity	1.009 (1.005, 1.014)
- ≥1.020, n (%)	43 (12 %)
Post-shift urine specific gravity	1.004 (1.002, 1.010)
- ≥1.020, n (%)	27 (7 %)
Pre-shift urine osmolality, mmol/kg	339 (208, 500)
Post-shift urine osmolality, mmol/kg	169.5 (109, 362)
Pre-shift serum osmolality, mmol/kg	283 (280, 286)
Post-shift serum osmolality, mmol/kg	279 (274, 284)
Pre-shift blood uric acid, mg/dL	5.52 (4.80, 6.33)
Post-shift blood uric acid, mg/dL	5.42 (4.71, 6.25)
Post-shift fractional excretion of uric acid, %	6.93 (5.25, 9.04)
- ≥ 10.0 %, n (%)	59 (16.3 %)
Pre-shift blood sodium, mEq/L	140 (138, 141)
- Hyponatremia (<135), n (%)	11 (3 %)
Post-shift blood sodium, mEq/L	137 (135, 139)
- Hyponatremia (<135), n (%)	78 (21 %)
Fractional excretion of sodium, %	1.03 (0.41, 1.80)
- ≥ 1.0 %, n (%)	184 (51 %)
Pre-shift creatine kinase, U/L	324.0 (232.5, 466.0)
Post-shift creatine kinase, U/L	448.0 (319.0, 602.0)
Dipstick post-shift acidic urine, pH = 5.0, n (%)	52 (14 %)
Dipstick post-shift leukocyte esterase, ≥70 + Leu/μL, n (%)	9 (2 %)
**Lifestyle Factors, n (%)**	
NSAID use*	13 (3.4 %)
Antipyretic use*	46 (12 %)
Currently smokes	24 (7 %)
**Markers of Kidney Function**	
Pre-shift serum creatinine, mg/dL	0.8 (0.7, 0.9)
Post-shift serum creatinine, mg/dL	0.9 (0.8, 1.1)
Change in serum creatinine, %	12.5 (0.0, 20.0)
Acute kidney injury** , n (%)	35 (9.5 %)
Pre-shift eGFR, ml/min/1.73 m^2^	120.1 (105.2, 127.5)
Post-shift eGFR, ml/min/1.73 m^2^	111.2 (93.2, 123.1)
Pre-shift blood urea nitrogen, mg/dL	11.0 (9.0, 15.0)
Pre-shift urine albumin-to-creatinine ratio	11.1 (6.5, 21.5)

eGFR: estimated glomerular filtration rate.

* Self-reported medication use in the last 24 hours. Non-steroidal anti-inflammatory drugs (NSAID) types included ibuprofen, aspirin, diclofenac, naproxen, or local brands that were identified as an NSAID. Antipyretics includes all NSAIDs plus acetaminophen brands.

** Acute kidney injury (AKI) is defined as an increase in post-shift creatinine by ≥ 0.3 mg/dl or to ≥ 1.5 times pre-shift.

**Table 3:** Regression analysis of workday practices and exposures with percent change in creatinine from pre- to post-shift among male sugarcane workers across two harvest seasons.

	Estimate (95 % CI), % ^A^	p-value*	Adjusted Estimate (95 % CI), % ^A^	p-value*

Work shift duration, per 1 hr	2.87 (−5.37, 0.38)	**0.02**	−2.71 (−5.79, 0.37)	0.08
Sugarcane cut, per ton^B^	2.22 (0.11, 4.33)	**0.04**	1.71 (−0.48, 3.9)	0.12
Rest breaks ^B^	1.86 (0.06, 3.66)	**0.04**	1.31 (−0.67, 3.29)	0.19
Number of electrolyte packets ^B^	−0.08 (−0.50, 0.34)	0.70	–	
Average heat index	1.33 (0.62, 2.03)	**<0.01**	1.17 (0.36, 1.97)	**<0.01**
Maximum heat index	0.17 (−0.22, 0.57)	0.39	–	
Particulate matter, PM_5_, per 1 μg/m^3^	−0.004 (−0.01, 0.003)	0.26		
Drinking water source ^B,C^				
- Municipality water (ref: well water)	−0.62 (−4.9, 3.66)	0.78	–	
- Other (ref: well water)	5.32 (−1.99, 12.62)	0.15	–	

A Coefficient is expressed as a percent (100 x beta estimate). Interpreted as a % change in creatinine from pre- to post-shift. Positive β estimate represents increases in creatinine across the shift. All models were adjusted for work group.

B Determined from self-report.

C Drinking water source for first 5-L water container fill-up.

* Bold values denote statistical significance at *p* < 0.05.

**Table 4:** Univariate regression analysis of factors with percent change in creatinine from pre- to post-shift among male sugarcane workers across two harvest seasons.

Daily Characteristics	Estimate (95 % CI), % ^A^	p-value*

**Demographics**		
Age	−0.08 (−0.26, 0.10)	0.34
Systolic blood pressure (per 10 mmHg)	−0.02 (−0.21, 0.17)	0.85
Diastolic blood pressure (per 10 mmHg)	−0.02 (−0.24, 0.21)	0.88
HbA1c* (per %)	0.59 (−1.43, 2.61)	0.57
Number of harvests worked (survey)	−0.09 (−0.29, 0.11)	0.35
Pre-harvest eGFR	0.11 (0.03, 0.20)	**0.009**
**Physiological Markers, Pre- and Post-Shift**		
Pre-shift serum osmolality	0.002 (−0.256, 0.259)	0.99
Post shift serum osmolality	0.072 (−0.059, 0.203)	0.28
Pre-shift urine osmolality	−0.002 (−0.01, 0.006)	0.58
Post shift urine osmolality	0.006 (−0.002, 0.013)	0.13
Pre-shift urine specific gravity	−0.03 (−2.73, 2.16)	0.82
Post-shift urine specific gravity	3.82 (1.51, 6.13)	**0.001**
Pre-shift blood sodium	0.691 (−0.012, 1.394)	**0.05**
Pre-shift fractional excretion of sodium	−1.51 (−3.44, 0.43)	0.13
Post-shift blood sodium	0.315 (−0.100, 0.729)	0.14
Post-shift fractional excretion of sodium	−2.03 (−3.31, −0.75)	**0.002**
Pre-shift blood uric acid	−1.06 (−2.366, 0.248)	0.11
Post-shift blood uric acid	2.40 (1.08, 3.71)	**0.0004**
Post-shift fraction excretion of uric acid	−0.56 (−1.02, −0.10)	**0.017**
Post-shift acidic urine		
- pH: 6.0–6.5 (ref: ≥7)	2.89 (−1.35, 7.13)	0.18
- pH: 5.0 (ref: ≥7)	3.55 (−1.09, 8.19)	0.13
Post-shift leukocyte esterase		
- ≥70 + Leu/μL (ref: < 70 + )	0.43 (−9.65, 10.51)	0.93
Pre-shift creatine kinase	−0.005 (−0.01, 0.001)	0.11
Post-shift creatine kinase	−0.001 (−0.005, 0.003)	0.52
Change in Creatine Kinase	0.003 (−0.005, 0.011)	0.45
Survey: Number of electrolyte packets	−0.483 (−2.68, 1.71)	0.66
**Core Body Temperature (CBT)**		
Area under the curve (AUC) CBT	−0.00002 (−0.00004, 0.000004)	0.10
CBT Categories	4.69 (0.63, 8.75)	**0.02**
- 38.0 – 38.4 °C	4.06 (−0.38, 8.50)	0.07
- 38.5 °C or greater	6.16 (0.18, 12.13)	**0.04**
Maximum CBT	5.73 (1.69, 9.78)	**0.006**
**Lifestyle Factors (survey)**		
Number of cigarettes since waking up	−0.19 (−3.96, 3.58)	0.91
Baseline Smoker		
- Current (ref: never)	3.22 (−3.71, 10.15)	0.35
- Former (ref: never)	0.17 (−6.72, 7.06)	0.96
NSAID use in last 24 h ^B^	−6.47 (−14.84, 1.91)	0.13
Antipyretic use ^B^	0.142 (−4.596, 4.88)	0.95

A Coefficient is expressed as a percent (100 x beta estimate). Interpreted as a % change in creatinine from pre- to post-shift. Positive β estimate represents increases in creatinine across the shift.

B Self-reported medication use in the last 24 h. NSAID types included ibuprofen, aspirin, diclofenac, naproxen, or local brands that were identified as an NSAID. Antipyretics includes all NSAIDs plus acetaminophen brands.

* Bold values denote statistical significance at *p* ≤ 0.05.

**Table 5:** Linear mixed effect models evaluating percent change in serum creatinine across the work shift among all participants.

Effect	Model 2: Estimate (95 % CI)^A^	p-value	Model 3: Estimate (95 % CI) ^A^	p-value

Age	−0.04 (−0.21, 0.14)	0.68	0.07 (−0.15, 0.29)	0.54
Pre-shift blood sodium	0.72 (0.02, 1.42)	0.04	0.63 (−0.29, 1.56)	0.18
Post-shift specific gravity (per 0.01)	3.38 (0.99, 5.76)	0.01	3.82 (0.84, 6.80)	0.01
Post-shift blood uric acid	2.11 (0.77, 3.45)	<0.01	2.46 (0.7, 4.21)	<0.01
Average heat index	1.21 (0.59, 1.82)	<0.01	1.33 (0.53, 2.14)	<0.01
Core Body Temperature Categories				
38.0 – 38.4 °C	–		2.30 (−2.13, 6.72)	0.30
38.5 °C or greater	–		8.95 (2.89, 15.02)	<0.01

A Coefficient is expressed as a percent (100 x beta estimate). Interpreted as a % change in creatinine from pre- to post-shift. Positive β estimate represents increases in creatinine across the shift.

## Data Availability

Data will be made available on request.
